# Steviol Represses Glucose Metabolism and Translation Initiation in Pancreatic Cancer Cells

**DOI:** 10.3390/biomedicines9121814

**Published:** 2021-12-02

**Authors:** Sonam Kumari, Mohammed Sikander, Shabnam Malik, Manish K. Tripathi, Bilal B. Hafeez, Murali M. Yallapu, Subhash C. Chauhan, Sheema Khan, Meena Jaggi

**Affiliations:** 1 Department of Pharmaceutical Sciences, University of Tennessee Health Science Center, Memphis, TN 38163, USA; sonam.kumari@nih.gov (S.K.); mohammed.sikander@utrgv.edu (M.S.); fnu.shabnam@utrgv.edu (S.M.); manish.tripathi@utrgv.edu (M.K.T.); bilal.hafeez@utrgv.edu (B.B.H.); murali.yallapu@utrgv.edu (M.M.Y.); subhash.chauhan@utrgv.edu (S.C.C.); sheema.khan@utrgv.edu (S.K.); 2 Department of Immunology and Microbiology, School of Medicine, University of Texas Rio Grande Valley, McAllen, TX 78504, USA; 3 South Texas Center of Excellence in Cancer Research, School of Medicine, University of Texas Rio Grande Valley, McAllen, TX 78504, USA

**Keywords:** steviol, pancreatic cancer, glucose metabolism, translation initiation

## Abstract

Pancreatic cancer has the worst prognosis and lowest survival rate among all cancers. Pancreatic cancer cells are highly metabolically active and typically reprogrammed for aberrant glucose metabolism; thus they respond poorly to therapeutic modalities. It is highly imperative to understand mechanisms that are responsible for high glucose metabolism and identify natural/synthetic agents that can repress glucose metabolic machinery in pancreatic cancer cells, to improve the therapeutic outcomes/management of pancreatic cancer patients. We have identified a glycoside, steviol that effectively represses glucose consumption in pancreatic cancer cells *via* the inhibition of the translation initiation machinery of the molecular components. Herein, we report that steviol effectively inhibits the glucose uptake and lactate production in pancreatic cancer cells (AsPC1 and HPAF-II). The growth, colonization, and invasion characteristics of pancreatic cancer cells were also determined by in vitro functional assay. Steviol treatment also inhibited the tumorigenic and metastatic potential of human pancreatic cancer cells by inducing apoptosis and cell cycle arrest in the G1/M phase. The metabolic shift by steviol was mediated through the repression of the phosphorylation of mTOR and translation initiation proteins (4E-BP1, eIF4e, eIF4B, and eIF4G). Overall, the results of this study suggest that steviol can effectively suppress the glucose metabolism and translation initiation in pancreatic cancer cells to mitigate their aggressiveness. This study might help in the design of newer combination therapeutic strategies for pancreatic cancer treatment.

## 1. Introduction

Pancreatic cancer (PanCa) is one of the most dangerous malignancies and is the source of numerous deaths worldwide [1]. In addition to the lack of early diagnosis, the highly metabolically active nature of pancreatic tumors leads to poor therapeutic outcomes, chemo-resistance, metastatic phenotype, and aggressive behavior of the disease [2]. Therefore, it is essential to investigate the underlying molecular mechanisms that are associated with aberrant tumor metabolisms and identify natural/synthetic molecules that can reduce metabolic behaviors of the pancreatic tumors/tumor cells. These studies may be highly useful for improving the survival rate of pancreatic cancer patients.

**Figure 1:**
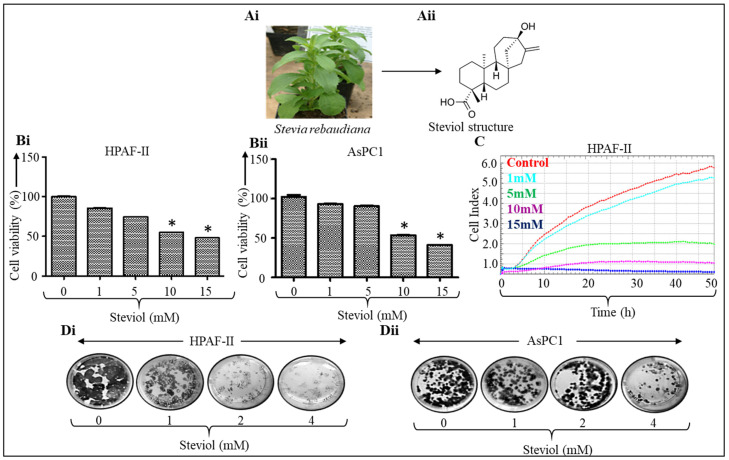
Steviol inhibits cell proliferation and clonogenic potential of pancreatic cancer cells. (**Ai**) A *Stevia rebaudiana* plant (www.wikipedia.com; accessed on 7 September 2021); (**Aii**) chemical structure of steviol; (**B**) pancreatic cancer cells were treated with different concentrations of steviol (0, 1, 5, 10, and 15 mM) and incubated for 48 h. Cell proliferation assay was terminated after addition of MTT reagent and recording the absorbance in HPAF-II (**Bi**) and AsPC1 cells (**Bii**); *n* = 3; * *p* < 0.05. (**C**) The cells were subjected to the xCELLigence instrument and cell proliferation assay (0, 1, 5, 10, and 15 mM of steviol) was conducted on the E-plate; (**D**) colony formation assay was performed on pancreatic cancer cells HPAF-II (**Di**) and AsPC1 (**Dii**). The cells were seeded, and after 4 days, treatment with steviol was performed at different concentrations (0, 1, 2, and 4 mM), and the cells were incubated for another 10 days.

In this study, we have identified steviol, a colonic metabolite of the natural sweetener steviol glycoside, which can effectively alter the glucose metabolism of pancreatic cancer cells. Steviol glycoside (stevioside) is obtained from the leaves of the *Stevia rebaudiana* plant (Figure 1Ai). It has a low calorie content and is 300 times sweeter than sucrose. Studies have shown that stevioside is very effective in reducing high glucose levels and inflammation. Reports also suggest that it has antioxidant and anti-cancer properties [3,4,5,6,7]. The metabolism of steviol begins in the human gut when stevioside (the precursor to steviol) is converted to steviol 16,17α-epoxide, and then to steviol through the action of bacteria present in the gut [8] (Figure 1Aii). After the completion of the metabolic process, it is relatively easier for steviol to be absorbed in the blood following oral administration.

Steviol is used as a food additive and is commercialized as stevia. It suppresses inflammation in the body and helps to regulate blood sugar, but studies on its benefits in humans are remain unclear [9,10,11,12]. Studies show that steviol treatment inhibited the growth and proliferation of colonic [13] and and breast cancer cells [14] and significantly reduced the promotion and initiation of lymphoblastoid cells [10].

Pancreatic tumors are characterized by having a highly tumor-supportive microenvironment, which favors biochemical and metabolic alterations and results in therapeutic resistance and aggressive tumor growth. Cancer cells undergo dysregulated glucose metabolism to sustain growth and proliferation rapidly. This also involves the upregulation of tumor-promoting genes [15]. In comparison to the quiescent cells, the cancerous cells do not use oxidative phosphorylation; they instead utilize the aerobic glycolysis mechanism (Warburg effect) [16], which in turn secretes lactate, which is responsible for generating an acidic microenvironment within tumors [17]. Alterations in metabolic pathways, which are governed by the involvement of increased glucose uptake and its conversion to lactate, are highly responsible for driving pancreatic cancer translational machinery and lead to aggressive metastatic tumors [18]. In addition, the chemo-resistant tumor cells need more glucose for their survival and continuous growth. Therefore, novel therapeutic strategies targeting glucose metabolism and the translation machinery are highly desired [19].

Different pathways can drive cancer aggressiveness and lead to metastasis [20]. Many molecules involved in translation are regulated through signaling events that require the mammalian target of rapamycin (mTOR). These include eukaryotic initiation factor 4E-binding proteins (4E-BPs) and S6 protein kinases, which are both repressors of translation proteins. In pancreatic cancer, the positive regulation of mTOR signaling hypophosphorylates 4E-BPs and activates S6 kinases, which leads to the initiation of translation process [21]. However, the molecular mechanisms by which amino acids modulate mTOR signaling remain unclear. Protein synthesis requires the cell’s metabolic energy and glucose as a fuel in high amounts, which is an important driver of mTOR pathway in cancer. 4E-BP regulates eIF4E to form the cap-binding complex, the Eukaryotic initiation factor 4F (eIF4F). The hypophosphorylation of 4EBP1 sequestrates eIF4E and prevents the interaction with eIF4G, thereby inhibiting translation process [21]. 4E-BP proteins and eIF4G share the competitive binding site to eIF4E. Furthermore, 4EBP1 works as an inhibitor of the cap-dependent translation. Translation-related proteins are mostly abnormal in tumors, such as mTOR and eIF4F, and result in the malignant phenotype, including immune escape, apoptosis inhibition, and energetic metabolism deregulating. Chemotherapeutic drugs targeting protein translation machinery to overcome cancer demonstrate improved outcomes in several cancer types in clinical trials [22] as the development of therapeutic agents that disrupt translation can control proliferation, drug resistance, and metastasis.

Therefore, targeting these molecules could be important for cancer treatment. One of the biggest challenges in pancreatic cancer therapeutics is preventing tumor relapse [23]. Following tumor resection, pancreatic cells being highly aggressive; the reviving of even a single cell can result in the recurrence of a tumor in the pancreas within a very short time [24]. In addition to the chemotherapies that follow surgical tumor resection, continued management of pancreatic tumor patients is desperately required to prevent tumor relapse in patients [25]. Given the scenario, therapeutic interventions using natural dietary constituents would be the best option as the possibility of toxicity or serious side effects will be comparatively minimal [26]. Therefore, this study investigates steviol for its ability to target pancreatic tumors and the regulatory proteins that govern protein translation, tumor proliferation, aggressiveness, and improve survival. Furthermore, we sought to understand the anticancer effects of steviol in pancreatic cancer cells and uncover its mechanism of action in pancreatic cancer. Our findings suggest that steviol modulating proliferation in pancreatic cancer cells may be attributed to its role in regulating glucose metabolism and protein translation and that it may act as a new treatment paradigm for pancreatic cancer management in patients. Overall, this study illustrates that steviol could be a promising candidate for therapeutic intervention, either alone or in combination strategies.

## 2. Materials and Methods

### 2.1. Cell Culture

The human pancreatic cancer cells (HPAF-II and AsPC1) used for this study were obtained from ATCC culture collection. These cells were maintained in DMEM/F12 or RPMI media, respectively, containing 10% fetal bovine serum which was supplemented with 10% FBS (Atlanta Biologicals, Flowery Branch, GA, USA) and antibiotic/antimycotic solution at 37 °C in a humidified atmosphere (5% CO_2_ and 95% air atmosphere).

### 2.2. Antibodies

Primary antibodies from Cell Signaling Technology were used to analyze the expression of important proteins, Bcl-2 (catalog #4223S), Bax (catalog #2772S), Bcl-xL (catalog #2764S), eIF4e (catalog #9742S), peIF4e (catalog #9741S), 4EBP1 (catalog #9452S), p4EBP1 (catalog #2855S), mTOR (catalog #4517S), *p*-mTOR (catalog #5536S), eIF4B (catalog #3592S), peIF4B (catalog #3591S), eIF4G (catalog #2498S), peIF4G (catalog #2441S), p18 (catalog # 2896S) and p21 (catalog #2947S). Cleaved PARP (sc-8007) and Cyclin D1 (sc-753) antibodies were obtained from Santa Cruz Biotechnology (Dallas, TX, USA). β-actin antibody was obtained from Sigma (catalog #A2228; St. Louis, MO, USA). The horseradish peroxidase conjugated rabbit (catalog #4011) and mouse (catalog #4021) secondary antibodies were procured from Promega (Madison, WI, USA).

### 2.3. Proliferation Assay

Pancreatic cancer cell lines were seeded at 5000 per well in 96-well plates and allowed to attach overnight. The following day, cells were treated with different concentrations of steviol for 48 h. MTT assays were performed to determine the antiproliferative potential of steviol on pancreatic cancer cells. Assays were terminated by adding 20 µL of MTT reagent to the cells. After 2 h of incubation, 100 µL of DMSO was added to the cells, then the plates were kept on a shaker for 10 min and the absorbance was recorded at 570 nm.

### 2.4. Western Blotting

Cell lysates were prepared and immunoblotting was performed [27]. Control and steviol-treated samples were loaded on a gradient gel (4–20%), transferred onto a PVDF membrane, and immunoblotted with respective antibodies after blocking in 5% milk. The secondary antibodies were added after washing the membranes with 1X TBST buffer, and the blots were developed with an ECL solution on a UVP machine.

### 2.5. Clonogenic Assay

Cells (250) were seeded in 12-well plates and grown for 4 days to form colonies [28,29]. The treatment was performed as soon as colonies started to appear. The experiment was terminated following 15 days of treatment and after washing, fixing with cold methanol, cells were stained with crystal violet and imaged. 

### 2.6. Real-Time xCELLigence Assays

To analyze the cell growth and migration inhibitory effects of steviol on pancreatic cancer cells, real-time proliferation and migration assays were conducted with the help of a xCELLigence instrument as described in our recently published studies [30]. Cells were seeded on the respective plates and were incubated in a 37 °C incubator with 5% CO_2_ condition. The experiment was terminated at the indicated time points and the results were analyzed.

### 2.7. Migration Assay

Cell migration assay was performed using Boyden chamber assay [31]. Cells (5 × 10^4^/well) were seeded in a 96-well plate on the upper chamber with media devoid of serum while the lower chamber contained 250 µL of media with serum. The cells were treated with steviol at different concentrations for 18 h and then fixed with 4% paraformaldehyde, followed by staining using crystal violet reagent. Thereafter, the plate was washed using tap water, air dried, and the images were captured for data analysis/quantification.

### 2.8. Invasion Assay

The cell invasion assay was conducted using Matrigel Invasion Chambers obtained from BD Biosciences as described earlier [31]. The chambers were hydrated for 2 h in serum-free media. Furthermore, the cells were seeded (25 × 10^4^/chamber) in 500 µL of serum-free media. The next day, the cells were treated with steviol at various concentrations in the chamber for 24 h. Cells were fixed using cold methanol, and staining was performed using crystal violet reagent. After washing and air-drying, images were captured for data analysis.

### 2.9. Cell Cycle Studies

This assay was performed through the Telford method as described earlier [32]. Briefly, cells were seeded (0.5 × 10^6^) in a 6-well plates, incubated overnight, and then treated with steviol for 12 h and 24 h. The cells were trypsinized, washed with PBS, and fixed with 70% ethanol. Furthermore, cells were incubated at 4 °C for 1 h and later stained with propidium iodide (PI) for about 4 h in the dark (at 4 °C). Cell-cycle analyses were performed in FL2 channel using a flow cytometer and ModFit analysis software.

### 2.10. Lactate Assay/ Glucose Assay

Both assays were conducted as described in our previous publication [27]. Cells were treated at described concentrations of steviol and glucose; lactate assays were conducted following the instructions mentioned in the kits (Cayman Chemicals, Ann Arbor, MI, USA). Thereafter, the absorbance readings were recorded, followed by plotting of the respective graphs based on the calculations.

### 2.11. Statistical Analysis

The statistical analysis for this manuscript was conducted by Student’s *t*-test. The *p*-values < 0.05 were regarded as statistically significant.

## 3. Results

### 3.1. Growth Inhibitory Effects of Steviol in Pancreatic Cancer Cells

To investigate the anti-tumorigenic properties of steviol, functional assays were performed in HPAF-II and AsPC1 pancreatic cancer cells. Cells were treated with steviol at various concentrations for 48 h. Both cell lines showed an inhibition of cell proliferation in a dose-dependent manner, with a significant inhibitory effect at 10 mM (HPAF-II; control vs. steviol 10 mM: 100 ± 1.57 vs. 54.8 ± 0.5 and AsPC1: 102.36 ± 3.91 vs. 53.54 ± 0.33) and 15 mM doses (HPAF-II; control vs. steviol 15 mM: 100 ± 1.57 vs. 48.05 ± 0.38 and AsPC1: 102.36 ± 3.91 vs. 40.9 ± 0.57) (Figure 1Bi-ii). Further, the suppression of proliferation in HPAF-II cells was also evaluated by xCELLigence assay after the treatment with steviol (Figure 1C). In addition, colony formation assays were performed to assess the long-term effect of steviol. The ability to form colonies is a crucial characteristics of cancer cells. The colony forming abilities of pancreatic cancer cells (AsPC1 and HPAF-II cells) were significantly suppressed by steviol as compared to the untreated cells (Figure 1Di-ii). These results demonstrated that steviol efficiently inhibited the growth and clonogenicity of pancreatic cancer cells.

### 3.2. Steviol Suppresses Migration and Invasion Capabilities of Pancreatic Cancer Cells

**Figure 2:**
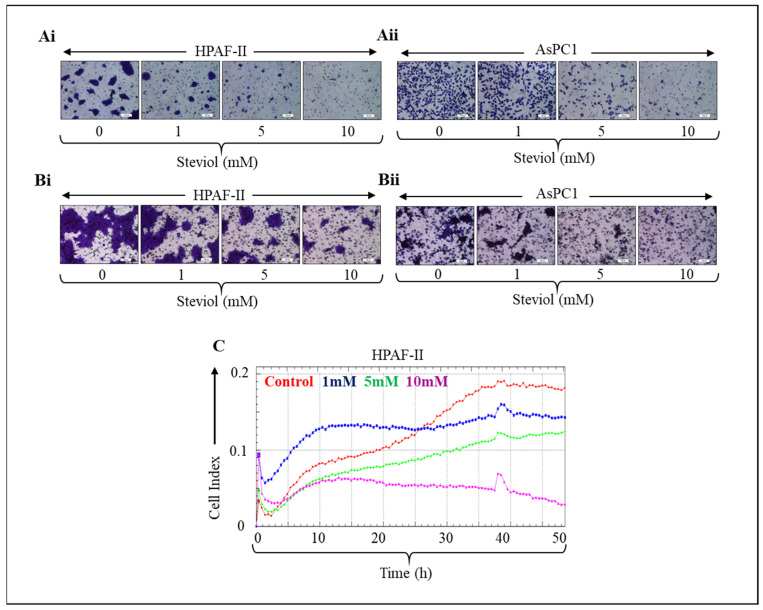
Steviol inhibits cell invasion and cell migration in pancreatic cancer cells. (**A**) Matrigel invasion assay was performed on pancreatic cancer cells HPAF-II (**Ai**) and AsPC1 (**Aii**); the cells were treated with steviol at indicated concentrations for 24 h. Thereafter, the cells were washed with PBS, fixed with methanol and stained using crystal violet. Upon drying, images were taken. (Magnification, 200×); (**B**) Boyden chamber migration assay was performed on pancreatic cancer cell cells HPAF-II (**Bi**) and AsPC1 (**Bii**); the cells were treated with 0, 1, 5, and 10 mM concentrations for 18 h. After that, cells were fixed with 4% paraformaldehyde, stained with crystal violet and left for drying. Furthermore, the images were captured (Magnification, 200×). (**C**) The pancreatic cancer cells were subjected to the xCELLigence instrument; cell migration assay (with 0, 1, 5, and 10 mM of steviol) was also carried out on the CIM-plate.

Pancreatic cancer cells were treated with steviol at various concentrations, and it was observed that steviol efficiently inhibited the cell invasion (Figure 2Ai-ii) and cell migration (Figure 2Bi-ii) abilities in a dose-dependent manner. Moreover, the suppression of migration in HPAF-II cells was evaluated by xCELLigence assay after treatment with steviol. This also demonstrated that steviol is effective in repressing the cell invasion and migratory potential of pancreatic cancer cells (Figure 2C).

### 3.3. Steviol Regulates Cell Cycle Machinery

**Figure 3:**
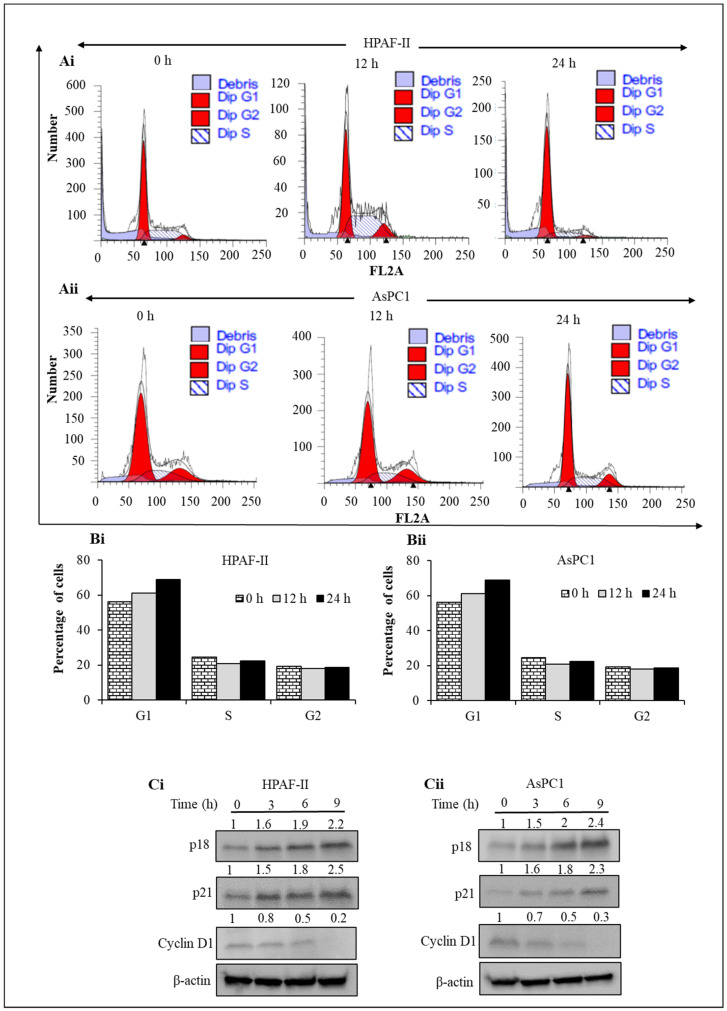
Steviol arrests G1 phase of cell-cycle distribution in pancreatic cancer cells. (**A**,**B**) Pancreatic cancer cells were seeded and treated with steviol at different time intervals (0, 12, and 24 h). Cell cycle experiment was performed to determine the effect of steviol (10 mM) on G1 cell cycle arrest in HPAF-II (**Ai**,**Bi**) and AsPC1 cells (**Aii**,**Bii**); (**C**) steviol modulates the key proteins involved in G1 phase of cell cycle arrest. Pancreatic cancer cells were treated with steviol (10 mM) at different time points (0, 3, 6, and 9 h). Whole-cell lysates were prepared and subjected to Western blotting. The expression level of important cell cycle proteins (*p* 18, *p* 21, and Cyclin D1) were evaluated in HPAF-II (**Ci**) and AsPC1 cells (**Cii**); the numbers over the blots represent the relative quantification of band intensities with the internal control (β-actin).

Regulation of the cell cycle is an important mechanism of many drugs. Therefore, to investigate the effect of steviol on cell-cycle regulation, pancreatic cancer cells were treated with steviol for different time points (0, 12, and 24 h) for cell-cycle analyses. It was observed that steviol induced G1 arrest, as depicted through the histogram graphs. The quantitative G1 population with respect to time is represented in HPAF-II and AsPC1 cells (Figure 3A,B). The proportion of G1 cells increased by about 27% in HPAF-II cells, and around 18% in AsPC1 cells after 24 h of steviol treatment. Furthermore, to understand the underlying mechanism of the G1 phase arrest, the regulatory proteins that are associated with the G1 stage of cell cycle were evaluated to determine if steviol induced any inhibitory effects on them. Interestingly, the immunoblotting results demonstrated that the inhibitory proteins associated with cell cycle, mainly *p* 18 and *p* 21, were significantly increased upon steviol treatment. On the other hand, Cyclin D1, which is critically involved in the progression of cell cycle [33], was remarkably downregulated in the presence of steviol in comparison to the untreated/control cells. These observations suggest that steviol treatment can regulate the cell cycle via the modulation of cycle-associated proteins (Figure 3Ci,ii).

### 3.4. Steviol Induces Apoptosis in Pancreatic Cancer Cells

**Figure 4:**
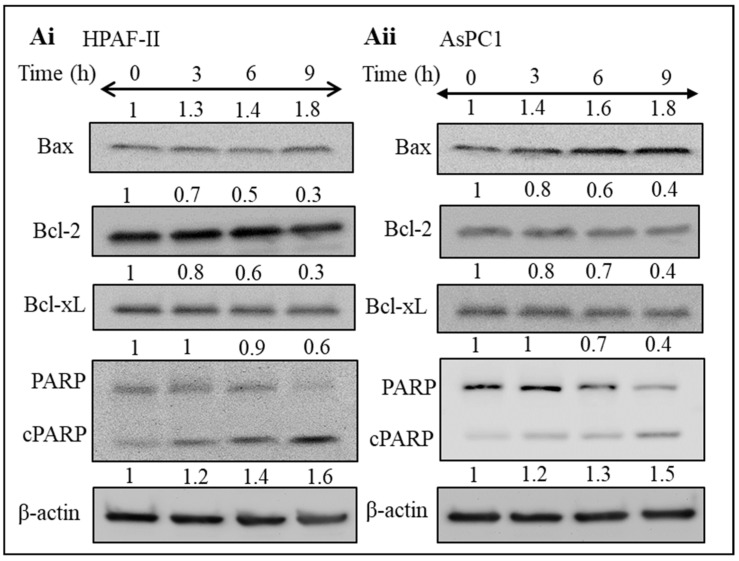
Steviol inhibits apoptosis in pancreatic cancer cells. (**A**) The expression level of important proteins associated with apoptotic pathway was evaluated in HPAF-II (**Ai**) and AsPC1 cells (**Aii**) of pancreatic cancer. Pancreatic cancer cells were treated with steviol (10 mM) at different time points (0, 3, 6, and 9 h), and Western blotting was conducted after preparation of cell lysates. Quantification of blots with respect to the loading control (β-actin) was performed as depicted by the numbers above the bands.

Apoptosis is commonly referred to as programmed cell death; cancer cells typically evade this process very effectively for acquiring uncontrolled proliferation and growth [34]. Several proteins are associated with the apoptotic pathway. Bax is one of the pro-apoptotic proteins crucially involved in this pathway. Moreover, other proteins such as Bcl-2, PARP, and Bcl-xL are the significant anti-apoptotic proteins regulating these processes [35]. It was interesting to evaluate whether steviol can modulate the apoptosis in pancreatic cancer cells. Therefore, the expression of apoptotic proteins was examined in pancreatic cancer cells after the treatment with steviol in a time-dependent manner (0, 3, 6, and 9 h). It was observed that the expression of Bax protein (pro-apoptotic) was upregulated in both HPAF-II and AsPC1 cell lines while the expression of important anti-apoptotic proteins (Bcl-2 and Bcl-xL) decreased significantly in a dose-dependent manner. In addition, increased PARP cleavage (a marker for early apoptosis) was observed after the treatment with steviol. These results collectively indicated that steviol facilitates the apoptosis process in pancreatic cancer cells (Figure 4Ai,ii).

### 3.5. Steviol Suppresses Translation Initiation in Pancreatic Cancer Cells

**Figure 5:**
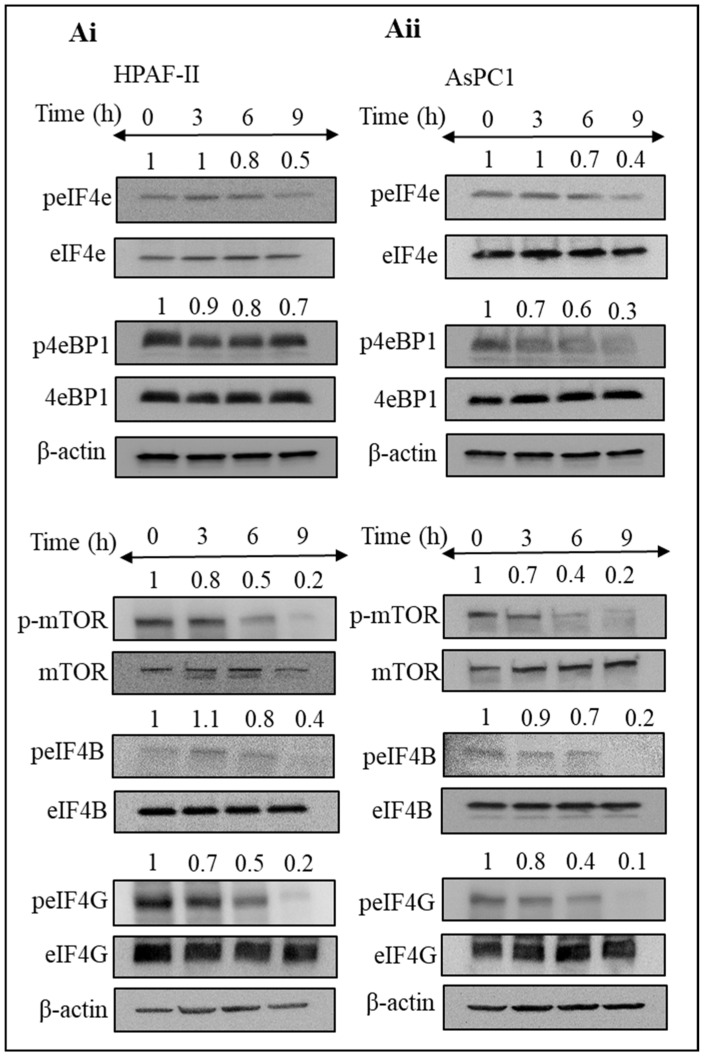
Steviol impedes translation initiation in pancreatic cancer cells. (**A**) Western blotting was performed to analyze the expression level of proteins associated with translation initiation in pancreatic cancer HPAF-II (**Ai**) and AsPC1 cells (**Aii**). Cells were treated with steviol (10 mM) at different time intervals (0, 3, 6, and 9 h), and whole cell lysates were prepared. The numbers above the blots represent the relative band intensity after normalizing with β-actin.

Disruption in the translation initiation machinery directly relates to carcinogenesis, and various oncogenic molecules and tumor suppressors play a major role in this process [36]. Therefore, it was imperative to understand the mechanism of action of steviol in relation to the translation initiation pathways. The expression of proteins associated with translation was analyzed, after the treatment of pancreatic cancer cells with steviol at different time intervals. Interestingly, we observed a remarkable inhibitory effect of steviol on the expression of proteins (peIF4E, peIF4B, peIF4G, p4EBP1, and *p*-mTOR) that are involved in the process of translation initiation (Figure 5Ai,ii). These proteins are required for the proper functioning of the translation initiation process. Steviol treatment clearly inhibited the expression of these essential proteins, which in turn hampered the formation of new proteins. It has been documented that activation of eIF4E increases the key tumorigenic mRNAs translation in order to enhance oncogenic characteristics [37], and steviol effectively decreased its phosphorylation. This suggest that steviol might delay the translation initiation process, which in turn reduces the tumorigenic activity of pancreatic cancer cells.

### 3.6. Steviol Decreases Glucose Uptake and Lactate Secretion in Pancreatic Cancer Cells

**Figure 6:**
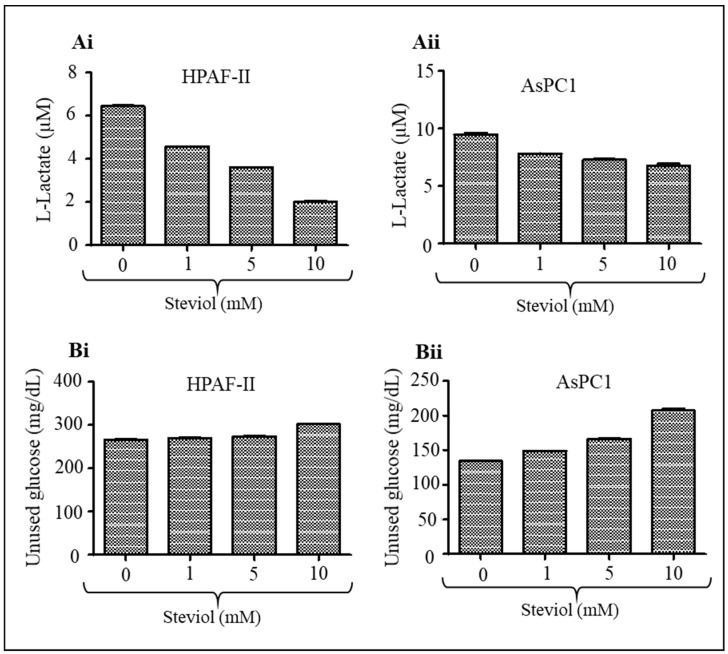
Steviol inhibits lactate secretion and glucose uptake in pancreatic cancer cells. (**A**) Effect of steviol on lactate secretion by pancreatic cancer cells as determined by glycolysis cell-based assay kit (Cayman Chemical, Ann Arbor, MI, USA). Bar graphs represent lactate secretion by HPAF-II (**Ai**) and AsPC1 (**Aii**) cells 24 h post-treatment of steviol. (**B**) Effect of steviol on glucose uptake by pancreatic cells as determined by glucose colorimetric assay kit after 48 h of steviol treatment (Cayman Chemicals, Ann Arbor, MI, USA). Bar graphs represent the unused glucose in HPAF-II (**Bi**) and AsPC1 (**Bii**) cells. *n* = 2; * *p* < 0.05.

Pancreatic cancer cells consume a very high level of glucose, which is metabolized by the glycolysis pathway, and produce lactate as the final product of glucose metabolism [27,38]. Therefore, we investigated if steviol could lower the glucose uptake and lactate production in pancreatic cancer cells. Indeed, steviol decreased lactate secretion in both HPAF-II (control vs. steviol 10 mM: 6.45 ± 0.05 µM vs. 2.01 ± 0.08 µM) and AsPC1 (9.49 ± 0.18 µM vs. 6.78 ± 0.26 µM) cell lines (Figure 6Ai-ii). Moreover, tumor cells undergo altered metabolism to support and sustain their upregulated growth and proliferative capabilities [27,38]. Studies have reported the potent anti-glycemic activity of steviol precursor in rats and humans [11,12]. Thus, we examined its effect on glucose uptake/total glucose levels in pancreatic cancer cells. Interestingly, it is observed that steviol treatment (10 mM) significantly (*p* < 0.05) reduced glucose uptake/consumption in pancreatic cancer cells (HPAF-II: 266.51 ± 1.0 mg/dL vs. 301.69 ± 1.65 mg/dL; and AsPC1: 134.21 ± 1.57 mg/dL vs. 207.14 ± 4.24 mg/dL) as compared to untreated control cells (Figure 6Bi-ii). These findings clearly suggest that steviol can be a useful compound to lower glucose metabolism and lactate production in pancreatic cancer cells.

## 4. Discussion

Pancreatic cancer is the most dreadful disease among various cancers, and its incidence/mortality rates are rising across the globe [39]. Pancreatic cancer cells are typically highly metabolically active [40]; as a result, the efficacy of current therapeutic options, including Gemcitabine (Gem), is very limited for the treatment of pancreatic cancer, currently the most widely used [41,42,43]. The Warburg effect supports increased glucose intake by pancreatic cancer cells to meet pathogenic requirements [19,27]. Several oncogenes and tumor suppressors have been shown to modulate metabolic reprogramming in pancreatic cancer including PI3K/Akt/mTOR pathways [44]. Accumulating studies have shown that these cell survival pathways are associated with translation initiation apparatus [45,46]. Hence, it is highly imperative to identify drugs/molecules that can effectively modulate metabolic reprogramming of pancreatic cancer cells. In this study, we have described a molecule, steviol, that can effectively alter metabolic activities and inhibit protein translation in pancreatic cancer cells.

Steviol is a natural compound with several beneficial properties including its sweet taste, low toxicity, and that is safe for human use. Since 2011, it has been approved for use in the food sector, and their safety has been assessed by several bodies, including the World Health Organization (WHO), and an ADI (Acceptable Daily Intake) [47,48]. Long-term human trials have been performed to assess the effect of orally administered stevioside at doses of 200–1500 mg/day [48]. Studies have also suggested its role as an anti-diabetic as well as anti-inflammatory, and anti-cancer roles [14,49]. Our studies demonstrate the growth inhibitory action of steviol in human pancreatic cancer (HPAF-II and AsPC1) cells via inducing apoptosis, as indicated by PARP cleavage and upregulation in Bax and inhibition in Bcl-2 and Bcl-xl. Steviol arrests cell-cycle progression in the G1 phase and lowers the glucose uptake/lactate production in pancreatic cancer cells. Additionally, steviol affected the expression of the key components of translation initiation machinery in pancreatic cancer cells (HPAF-II and AsPC1).

A growing body of evidence suggests that abnormal activation of translation initiation factors can result in malignant transformation and maintenance of altered phenotypes in several human cancers [50,51,52,53,54]. Increased signaling flux from eIF4F activation causes deregulated translation initiation, which is implicated in cancer initiation and progression [55,56]. It is observed that a number of angiogenesis factors and oncoproteins, as well as pro-survival proteins and proteins involved in cancer invasion and metastasis, are all found to be affected by increased assembly of the eIF4F translation initiation complex [57]. The eIF4E is required for the assembly of the eIF4F complex and is bound to the eIF4E binding proteins (4EBPs) under normal conditions.

**Figure 7:**
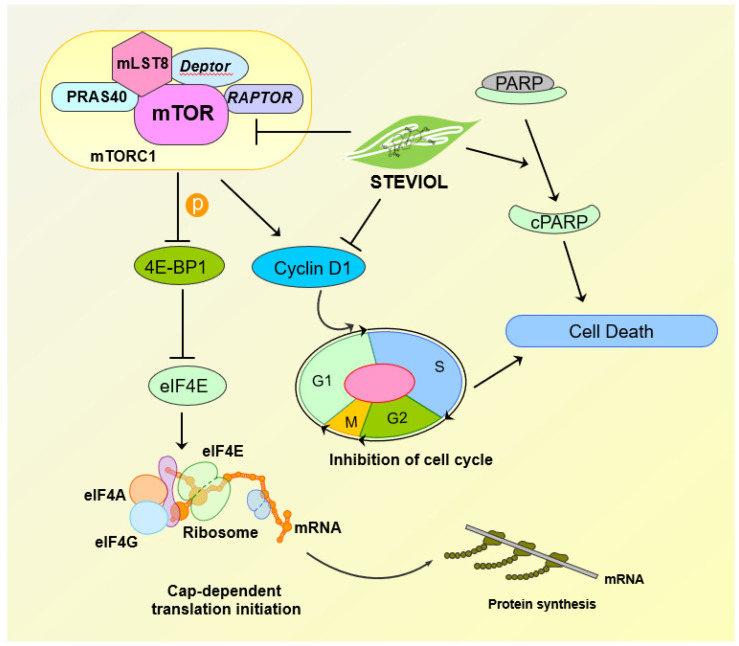
Schematic representation depicting mechanism of action of steviol in pancreatic cancer. The lines represent direct or indirect activation (arrow head) and inactivation (blunt end) by various signaling molecules.

The 4EBP1 protein suppresses eIF4F formation by competing with eIF4G for eIF4E binding. Abnormal eIF4E expression has been linked to cancer susceptibility and malignant transformation [58,59]. Therefore, targeting eIF4F translation initiation complex might be an ideal strategy for anti-cancer drug research for pancreatic cancer patients. Interestingly, steviol treatment increased the expression of p4EBP1 protein in pancreatic cancer cells. Therefore, we further sought to investigate the changes in expression level of the other key proteins that are involved in the translation initiation process. We observed repression of the key translation initiation proteins (peIF4E, peIF4B, peIF4G, and *p*-mTOR) in steviol-treated pancreatic cancer cells. These proteins are strongly associated with the translation-initiation process in eukaryotic cells [58,59]. Owing to their crucial roles in energy metabolism, which is an essential feature of pancreatic cancer cells, steviol treatment probably led to a decrease in the glucose uptake lactate secretion in pancreatic cancer cells. These results are consistent with the findings by Chen et al. (2016), who reported the growth of an inhibitory effect of steviolbioside in human pancreatic cancer cells [60]. Therefore, our findings delineate that steviol might be a potent compound that can induce a metabolic shift in pancreatic tumors (Figure 7). Although there are some concerns for its toxic effects on lymphocytes [61] and a lack of significant reduction of pancreatic acinar carcinoma development by stevia extract (0.02% *w/v*) in a murine model [62], more research into their efficacy and bioavailability is warranted.

In conclusion, this study has investigated a dietary constituent, steviol, for its ability to target pancreatic tumor and the regulatory proteins that govern protein translation and tumor proliferation and aggressiveness (Figure 7). Steviol, being safe for human use, can be considered as an effective modality for controlling glucose metabolism and regulating protein translation. Therefore, this study provides an important insight regarding management of pancreatic cancer patients to prevent relapse and recurrence of tumors. Additionally, it can help in improving therapeutic outcomes of frontline chemotherapeutic drugs when used in combination. This strategy can enhance the survival of pancreatic cancer patients; however, advanced clinical research in this direction is warranted to fully support this notion.

## Data Availability

The data presented in this study are contained within this article.
